# How Essential Is to Focus on Physician's Health and Burnout in Coronavirus (COVID-19) Pandemic?

**DOI:** 10.7759/cureus.7538

**Published:** 2020-04-04

**Authors:** Kaushal Shah, Gaurav Chaudhari, Dhwani Kamrai, Amindeep Lail, Rikinkumar S Patel

**Affiliations:** 1 Psychiatry, Griffin Memorial Hospital, Norman, USA; 2 Psychiatry, Johns Hopkins Bloomberg School of Public Health, Baltimore, USA

**Keywords:** burnout, physician burnout, resident burnout, covid-19, coronavirus, pandemic, psychiatry, ecfmg, gme, mental health

## Abstract

An infection of novel coronavirus (COVID-19) that originated from Wuhan city of China in December 2019 converted rapidly into pandemic by March 11, 2020. To date, the number of confirmed cases and deaths has risen exponentially in more than 200 countries, with an estimated crude mortality ratio of at least over 2%. The unpreparedness to tackle the unprecedented situation of coronavirus has contributed to the rising number of cases, which has generated an immense sense of fear and anxiety amongst the public. It has further resulted in the inadequacy and unavailability of essential medical supplies, physicians, and healthcare workers (HCW). Although the chief focus is on minimizing transmission through prevention, combating infection, and saving lives by ramping up the development of treatment and vaccines, very little attention is on the critical issue of physician burnout, resident burnout, and the psychological well-being of HCW. Until now, no significant steps have been taken by the authorities to minimize the COVID-19 specific contributing factors for burnout. The COVID-19 has posed strain on the entire healthcare system already, and it is vital to remediate the issue of physician and resident burnout urgently with concrete actions to avoid subsequent potential short-term and long-term adverse implications.

## Editorial

The novel coronavirus (COVID-19) initially identified in the Wuhan city of Hubei province of China in December 2019 has spread to more than 200 countries until now [1,2]. The World Health Organization officially declared it a pandemic on March 11, 2020, with the current confirmed cases and deaths across the globe of over 465,915 and 21,031, respectively [2]. A national emergency was declared in the United States on March 13, 2020, as it spread in all 50 states with the current tally at about 85,356 infected people and 1,246 deaths [1]. On March 18, 2020, the administration imposed the Defense Production Act to ramp up the production of necessary medical equipment and passed two trillion dollars of historic stimulus in the economy [3]. This rising number of cases, unpreparedness, lack of vital resources, excessive workload, and the inability to contain the spread has caused fear and anxiety amongst the public, including physicians, residents, fellows, and healthcare workers. 

In the United States, about 54.4% of physicians have shown at least one symptom of burnout in the form of emotional exhaustion, depersonalization, or reduced senses of accomplishment. A similar pattern at varying degrees is found in the residents and fellows. The phenomena of physician burnout are adequately studied and have a direct negative impact on fatigue, stress, anxiety, depression, mood disorders, substance abuse, suicides, poor patient quality care, early retirements, and unexpected resignations [4]. In addition to the known physician burnout contributors such as work factors (excessive workloads and work hours), personal characteristics (work-life imbalance, inadequate support, sleep deprivation), and organization factors (workload expectations, insufficient rewards, and interpersonal communication, negative leadership), it essential to identify and remediate outbreak specific issues to avoid the unwanted social, psychological, and economic burden [4,5]. 

Based on the research of past outbreaks of the severe acute respiratory syndrome (SARS), Middle East respiratory syndrome (MERS), influenza, and H1N1, it is well established that physicians, residents, fellows, and the healthcare workers experience a varying degree of burnout. Anxiety and stress developed in the physicians during the outbreaks found to have a positive correlation with Maslach burnout inventory scores [4,5]. Other factors identified in addition to usual contributors to burnout are lack of control over procedures, infection control measures, the false notion of safety precautions, poor communication and directives, lack of preparedness and emotional support, inadequate personal protective equipment (PPE), and perceived fatality [5].

Unfortunately, the issue of physician burnout is not investigated in totality to determine suitable interventions and effectiveness during the past outbreaks. We propose firm measures to address this deficit in no particular sequence. First, empower physicians by providing essential resources adequately in the form of PPE, beds, medicines, ventilators, educational guidelines, and research updates. Second, provide consistent and updated guidelines regularly to staff for managing patients through triage based on the case priority and severity. Third, recruit additional allied healthcare and administrative staff, including medical assistants, scribes, coordinators, clerical assistants, and triage specialists, to unburden clinicians to some extend from nonclinical tasks and medical notes. Fourth, increase the workforce of clinicians by hiring additional physicians, nurse practitioners (NP), and physician assistants (PA). Furthermore, provisionally ease the rules of mandatory oversight of NPs and PAs by physicians at the times of the high volume of cases. Fifth, loosen up the restrictions on the visa requiring US-licensed physicians and allow unmatched Educational Commission For Foreign Medical Graduates (ECFMG) certified physicians to assist at least in a limited demanded capacity. Sixth, extend the medical license that is set for renewal now and in the next six months. Seventh, initiate medical residency early for the matched candidates and allocate additional emergency budget for graduate medical education (GME) residency positions. Eight, facilitate the setup of telemedicine and telepsychiatry services to address the medical and psychiatric needs of the patients and staff. Ninth, provide support with clear communication from the leadership regarding quarantine directives, guidelines, and management protocol. Tenth, restrict excessive workload by scheduling breaks, limit work hours in emergency and intensive care units, and provide regular psychosocial support, essential basic needs, mindfulness sessions, and resilience training. Eleventh, ensure the safety and health of all staff members by the daily screening of vital signs, possible symptoms of infection, and signs of burnout. Twelfth, train, and leverage the expertise of the residents and fellows as a frontline worker to handle coronavirus cases or non-coronavirus cases. Thirteenth, protect, and support residents and fellows by creating an action plan and temporarily deferring the rules for GME training and board eligibility.

As physicians are the frontline healthcare workers in responding to the COVID-19 outbreak, it is of paramount importance that we invest immediately in the wellness of the physicians to avoid shortage due to burnout. We propose necessary basic measures for the authorities and organizations towards identifying and managing the physician burnout and resident burnout threats in the early stages of the COVID-19. It is in the best interest of public health to start acting on this dreadful issue now instead of reacting it later when it deepens further.
